# High-Precision Semiconductor Laser Current Drive and Temperature Control System Design

**DOI:** 10.3390/s22249989

**Published:** 2022-12-18

**Authors:** Yitao Zhao, Zengguo Tian, Xiangyu Feng, Zhengyuan Feng, Xuguang Zhu, Yiqun Zhou

**Affiliations:** 1 School of Physics and Microelectronics, Zhengzhou University, Zhengzhou 450001, China; 2 Luoyang Dejing Intelligent Technology Company Ltd., Luoyang 471032, China

**Keywords:** semiconductor laser, thermoelectric cooler, constant current driver circuit, temperature control, system identification, fuzzy PID

## Abstract

To solve the problem in which the output power and wavelength of semiconductor lasers in fiber optic sensing systems are easily affected by the drive current and temperature, a high-precision current drive and temperature control system was developed in this study. The embedded system was used to provide a stable drive current for the semiconductor laser through closed-loop negative feedback control; moreover, some measures, such as linear slow-start, current-limiting protection, and electrostatic protection, were adopted to ensure the stability and safety of the laser’s operation. A mathematical model of the temperature control system was constructed using mechanism analysis, and model identification was completed using the M sequence and differential evolution (DE) algorithms. Finally, the control rules of the fuzzy proportional integral differentiation (PID) algorithm were optimized through system simulation to make it more suitable for the temperature control system designed in this research, and the accurate control of the working temperature of the semiconductor laser was realized. Experimental results showed that the system could achieve a linearly adjustable drive current in the range of 0–100 mA, with an output current accuracy of 0.01 mA and a temperature control accuracy of up to 0.005 °C.

## 1. Introduction

Due to the advantages of semiconductor lasers, such as small size, light weight, high efficiency, and easy modulation [1,2], they have been widely used in precision measurements [3], environmental monitoring [4], gas detection [5], and other fields. In an optical-fiber sensing system, an external signal under testing changes the optical properties of the fiber, such as the power, wavelength, frequency, phase, and polarization state, according to certain physical laws; this signal is sensed by measuring the change in optical properties. This measurement type mainly involves a real-time modulation of the light propagation in optical fibers by the external signals to be measured. It can be classified into five types, depending on the change in the physical properties of the light waves modulated by external signals: intensity modulation, frequency modulation, wavelength modulation, phase modulation, and polarization state modulation [6]. As any small change in the light waves can directly affect the measurement results, there are strict requirements for the output optical power, central wavelength, and other output characteristics of semiconductor lasers in fiber-optic sensing systems. However, since semiconductor lasers are sensitive, changes in both their drive current and operating temperature can result in significant impacts on their output characteristics [7,8]. The typical optical power-current modulation rate, wavelength-current modulation rate, and wavelength-temperature modulation rate are 0.2 mW/mA, 0.01 nm/mA, and 0.1 nm/°C, respectively [9]. Moreover, semiconductor lasers are susceptible to damage, due to static electricity and inrush currents [10]. Thus, to ensure the detection accuracy and operational stability of fiber-optic sensing systems, it is essential to design a high-precision current drive and temperature control system.

Some researchers have conducted extensive research on the drive and temperature control systems of semiconductor lasers and have achieved a few results. For example, Zhang et al. [11] achieved a wide range of temperature control, from −12 to 120 °C, with the variable-domain PID control algorithm; however, its temperature control accuracy was only 0.1 °C. Cheng et al. [12] designed a temperature control system for semiconductor lasers based on an analog PID algorithm with a maximum error of 0.08 °C. Wang et al. [13] used an incremental PID algorithm to achieve a temperature control error of ~0.05 °C for a semiconductor laser. Moreover, a system designed by Luo et al. [14] achieved an adjustable drive current of 0–100 mA, with maximum relative errors of 0.06% for current control and 0.03 °C for temperature control. Through experimentation, Zhao et al. [15] found a certain sinusoidal fluctuation relationship between the output power of the laser and the working temperature of the laser. By analyzing the reasons for this phenomenon, the temperature control circuit with the ADN8830 chip as the core was improved so that the temperature control accuracy was increased from 0.5 °C to 0.02 °C. Gao [16] used a genetic algorithm to optimize the control parameters of the PID controller, in order to obtain optimal parameter values so that the temperature fluctuation range of the semiconductor laser was within 0.02 °C. Xin et al. [17] developed a double closed-loop temperature control system. The outer-loop temperature control was based on the integrated module MTD1020T as the core, and a temperature control accuracy of 0.5 °C could be achieved by optimizing the digital PID parameters; the inner-loop temperature control was based on the LTC1923 control chip as the core, and a temperature control accuracy of 0.01 °C could be achieved by adding the differential amplification link and setting the PI link. The experimental results showed that the achieved temperature control accuracy of the semiconductor laser was within 0.02 °C in 4 h. Su et al. [18] used a current series negative feedback circuit to achieve a continuously adjustable semiconductor laser drive current in the range of 0–100 mA; furthermore, the MAX1978 chip as the core with an analog PID circuit achieved 0.01 °C temperature control accuracy of the semiconductor laser. Chen et al. [19] used the MAX1978 chip to achieve a temperature control accuracy of 0.01 °C, and extended the operating linear region of the constant current source. Xu et al. [20] implemented the design of a dual-channel current source using a deep negative feedback circuit, which could provide a drive current with a regulation accuracy of 0.01 mA for a semiconductor laser. Through the unremitting efforts of researchers, the performance of the drive and temperature control system of semiconductor lasers has continuously improved. However, there is still room for further improvement with regard to the accuracy of current and temperature control, and there have not been enough studies on the construction of temperature control system mathematical models.

This study systematically introduces the design ideas for a semiconductor laser current drive and temperature control system. By studying constant-current drive circuits, laser protection circuits, laser temperature measurement and control systems, model constructions of temperature control systems, and the simulation and optimization of control algorithms, a high-precision semiconductor laser current drive and temperature control system was designed. The experimental results showed that the system designed in this study can provide a stable driving current for the semiconductor laser with a regulation accuracy of 0.01 mA, and can improve the temperature control accuracy of the semiconductor laser to 0.005 °C. This study’s mathematical model of temperature control system was constructed with high accuracy by a combination of mechanism analysis and system identification. This method has good applicability, and can provide a good reference for relevant researchers.

## 2. Characteristics of Semiconductor Lasers

A semiconductor laser is a miniaturized laser with a positive-negative (PN) junction or a positive-intrinsic-negative (PIN) junction as the working medium [21]. The threshold current, output power, and wavelength of semiconductor lasers all vary with temperature, due to the large effect of temperature on the physical properties of PN junctions [22,23]. In addition, the output power and wavelength of semiconductor lasers are directly related to their operating current [24]. To keep the output characteristics of semiconductor lasers stable, it is necessary to analyze their current and temperature characteristics.

### 2.1. Current Characteristics

**Figure 1:**
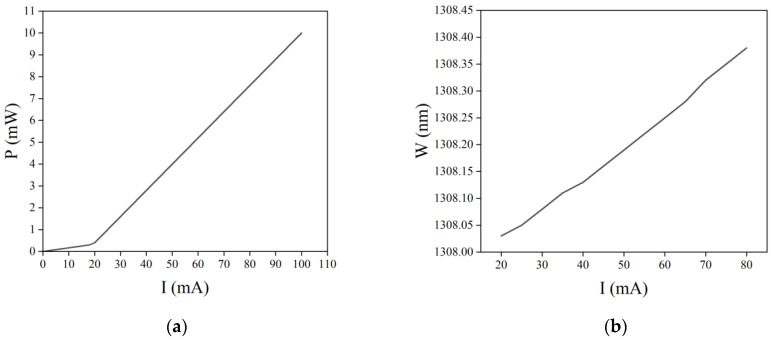
Current characteristics of semiconductor lasers: (**a**) P-I characteristic curve and (**b**) W-I characteristic curve.

The power-current (P-I) characteristics of semiconductor lasers are shown in Figure 1a. When the injection current of a semiconductor laser gradually increases, the output optical power slowly increases. However, when the injection current exceeds a certain value, the output optical power sharply increases. The current that corresponds to this inflection point is the threshold current of the semiconductor laser. Figure 1b shows the wavelength-current (W-I) characteristic curve of a semiconductor laser at 25 °C. It can be seen that as the drive current increases, the output wavelength of the semiconductor laser gradually moves toward the long wave direction. Therefore, the stable output optical power and wavelength of semiconductor lasers need to be achieved by precisely controlling their drive current.

### 2.2. Temperature Characteristics

**Figure 2:**
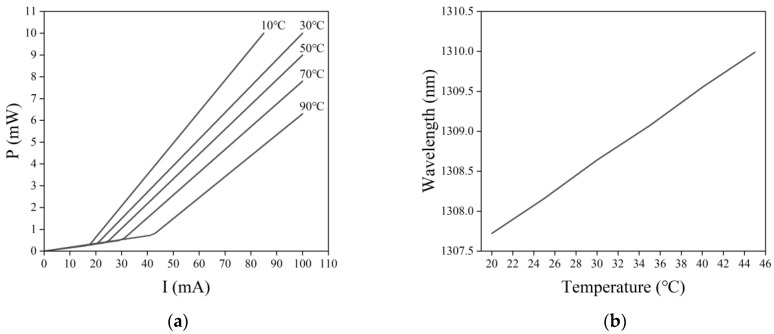
Temperature characteristics of semiconductor lasers. (**a**) Temperature effect on the P-I characteristic curve. (**b**) Temperature effect on the output wavelength.

It has been shown that the temperature effect on the operating performance and stability of semiconductor lasers is mainly reflected in the temperature effect on their wavelength, threshold current, output power, and service life [25]. The temperature characteristics of semiconductor lasers are shown in Figure 2. Due to the considerable current density and dissipated power density that the PN junction is subjected to internally, there are inevitably various nonradiative losses, free carrier absorption, and other loss mechanisms [26]. As a result, a significant portion of the used electrical energy is converted into heat, causing a rapid rise in the temperature of semiconductor lasers. When the temperature of a semiconductor laser increases, the output power decreases, and heat consumption increases, thus further increasing the laser’s temperature and resulting in a vicious circle. As shown in Figure 2a, with an increase in temperature, the inflection point of the P-I characteristic curve gradually moves to the right. Thus, the threshold current of semiconductor lasers increases with an increase in temperature. Moreover, when the temperature increases from 10 °C to 90 °C, the threshold current increases from 17 mA to 43 mA, and the output power of the semiconductor laser at the same operating current decreases with an increase in temperature. In addition, temperature changes cause changes in the forbidden bandwidth, with the active layer gap narrowing as the temperature increases, and the output wavelength shifting in the long-wave direction (i.e., producing a red shift) [27]. Figure 2b shows the temperature effect on the output wavelength of a semiconductor laser at a drive current of 46 mA. To improve the stability of the laser’s output optical power and wavelength, the laser operating temperature needs to be kept constant.

## 3. Current Drive and Temperature Control Circuit Design

### 3.1. Overall Design Scheme

**Figure 3:**
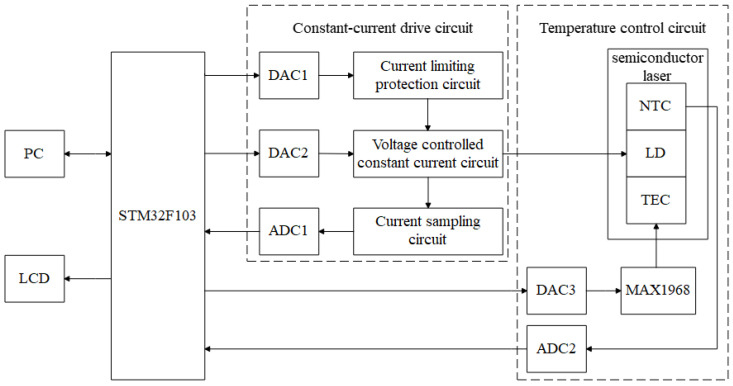
Overall system design.

As shown in Section 2, to ensure a stable wavelength and optical power output from a semiconductor laser, a stable drive current needs to be provided, and the operating temperature needs to be kept constant. Therefore, this study proposed a high-precision semiconductor laser current drive and temperature control system. The overall design scheme is shown in Figure 3, and it mainly includes three parts: a constant-current drive module, a temperature control module, and a microcontroller module. The constant-current driver module consists of a voltage-controlled constant-current circuit, a sampling feedback circuit, and a laser protection circuit to provide a stable and adjustable operating current for the laser. The temperature control module collects the operating temperature of the laser diode (LD) via a negative temperature coefficient (NTC) thermistor, and controls the operating temperature of the LD by regulating the current level and direction of the thermoelectric cooler (TEC) via a driver chip (MAX1968). The microcontroller module uses an embedded microprocessor (STM32F103), which is responsible for driving the digital-to-analog converter (DAC) and analog-to-digital converter (ADC) in the circuit module, and for completing the data processing and algorithmic operations in the system.

### 3.2. Constant-Current Driver Circuit Design

**Figure 4:**
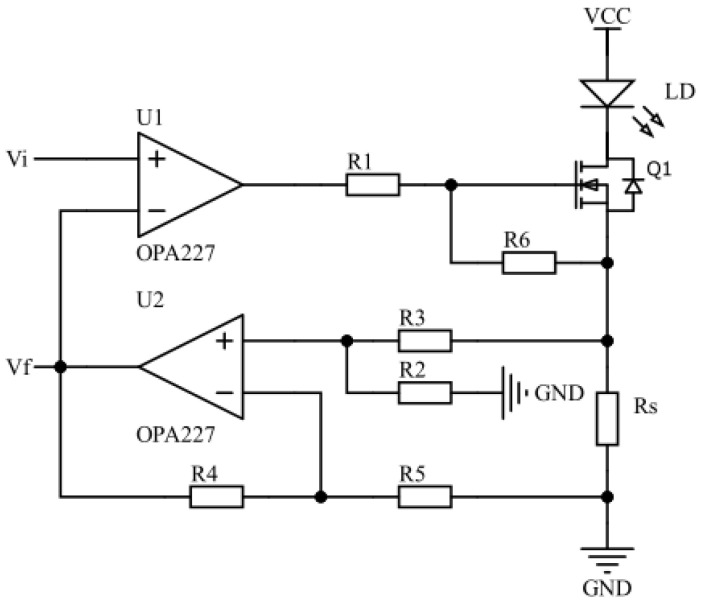
The constant-current driver circuit.

Using the negative feedback principle, the constant-current drive circuit controls the magnitude of the drive current by adjusting the level of conduction of the metal oxide semiconductor field effect transistor (MOSFET), in order to ensure the stability of the drive current. As shown in Figure 4, the drive current flows through the LD and generates a voltage drop across $R_{s}$. The amplifier circuit formed by operational amplifier U2 and resistors $R_{2}$-$R_{5}$ amplifies the voltage across resistor $R_{s}$ to build a feedback voltage $V_{f}$, and transmits $V_{f}$ to the inverted input of U1. The voltage at the positive phase input of U1 is the set voltage $V_{i}$. Moreover, the degree of MOSFET conduction is regulated by comparing the voltage magnitude of $V_{f}$ with that of $V_{i}$, thus controlling the amount of drive current flowing through the LD. The whole constant-current driver circuit has a simple structure, and contains four parts: a voltage reference source ($V_{i}$), error amplifier (U1), adjustment transistor (Q1), and a feedback loop (U2, $R_{s}$, $R_{2}$-$R_{5}$). The voltage reference source, i.e., the set voltage $V_{i}$, is provided by the DAC8830, and the operational amplifier U1 acts as an error amplifier, forming a control loop for the drive current with the adjustment transistor and the feedback loop. When the drive current fluctuates, the output voltage of the error amplifier changes accordingly, and the change in drive current is suppressed by the negative feedback regulation effect of the control loop. For example, when the drive current is decreased, the feedback voltage $V_{f}$ generated through the feedback loop is reduced as well. The reference voltage originates from the positive phase input of the error amplifier to provide a stable reference voltage. The decrease in $V_{f}$ will result in an increase in the output voltage of the error amplifier, and an increase in the gate voltage of the regulator tube Q1. The higher gate-source voltage difference of Q1 will cause an increase in the drain current, thus suppressing the decrease in drive current and maintaining a stable output.

The circuit in Figure 4 shows that when the drive current of LD is kept stable, the following equations apply:

(1) $$V_{U_{2+}}=\frac{R_{2}}{R_{2}+R_{3}}\cdot I_{LD}\cdot R_{s},$$

(2) $$V_{U_{2-}}=V_{U_{2+}},$$

(3) $$V_{f}=\left(1+\frac{R_{4}}{R_{5}}\right)\cdot V_{U_{2-}},$$

(4) $$V_{f}=V_{i}.$$

In Equations (1)–(4), $I_{LD}$ denotes the drive current flowing through the LD, and $V_{U_{2+}}$ and $V_{U_{2-}}$ denote the voltages at the forward and reverse inputs of the operational amplifier U2, respectively. The sampling resistor $R_{s}$ has a resistance of 1 Ω, $R_{2}$ = $R_{4}$ = 10 kΩ, and $R_{3}$ = $R_{5}$ = 1 kΩ. By combining Equations (1)–(4) and substituting the specific values of the relevant components for calculation, the drive current flowing through the LD can be obtained as follows:

(5) $$I_{LD}=\frac{V_{i}}{\left(1+\frac{R_{4}}{R_{5}}\right)\left(\frac{R_{2}}{R_{2}+R_{3}}\right)\cdot R_{s}}=\frac{V_{i}}{10R_{s}}.$$

From Equation (5), it can be seen that the drive current $I_{LD}$ of the LD shows an ideal linear relationship with the set voltage $V_{i}$. The 10 in the denominator of Equation (5) represents the amplification factor of the amplification circuit that contains operational amplifier U2 and resistors $R_{2}$-$R_{5}$. Without modifying the range of the set voltage $V_{i}$, the magnitude of enlargement can be changed by changing the resistance values of $R_{2}$-$R_{5}$; hence, the set range of the drive current can be tuned.

### 3.3. Current Limiting and Electrostatic Protection Circuit Design

**Figure 5:**
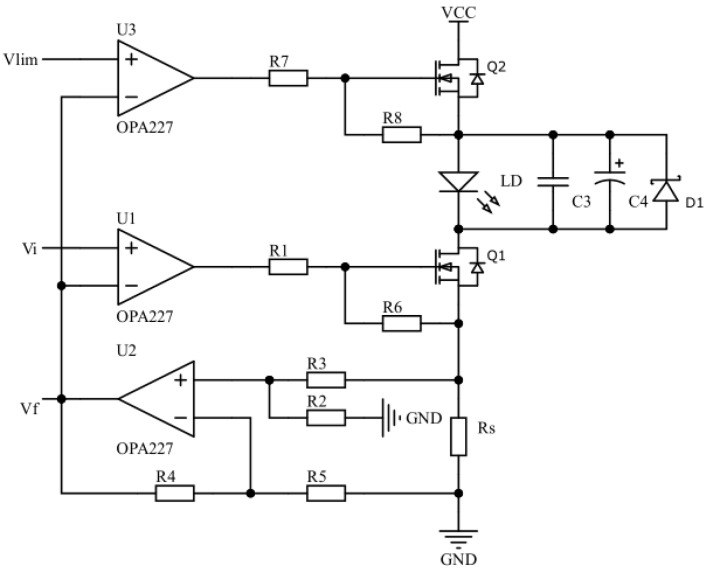
The constant-current driver circuit and the protection circuit.

Under practical operating conditions, excessive drive current and static electricity are important causes of damage to lasers, and can also shorten their service life [28]. The current limiting protection circuit for the laser was designed using the same operating principle as the constant-current driver circuit. The voltage at the positive-phase input of the operational amplifier U3 is the current-limiting voltage $V_{lim}$. When $V_{i}<V_{lim}$, the degree of conduction of the MOSFET Q1 is less than that of Q2, and the driving current is determined by Q1 and has a magnitude of $V_{i}/10$. When $V_{i}>V_{lim}$, the degree of conduction of the MOSFET Q1 is greater than that of Q2, and the size of the drive current in the drive circuit is clamped by Q2. At this time, the size of the current flowing through LD is $V_{lim}/10$. In addition, the small capacitance ceramic capacitor $C_{3}$, large capacitance electrolytic capacitor $C_{4}$, and transient diode $D_{1}$ are connected in parallel at both ends of the LD. Through $C_{3}$, the high-frequency noise carried by the circuit front is filtered out. The purpose of connecting $C_{4}$ in parallel is to limit the voltage mutation at both ends of the LD, and $D_{1}$ is used to protect the LD from damage due to static electricity. Figure 5 shows the specific circuit.

### 3.4. Linear Slow-Start Design of the Drive Circuit

**Figure 6:**
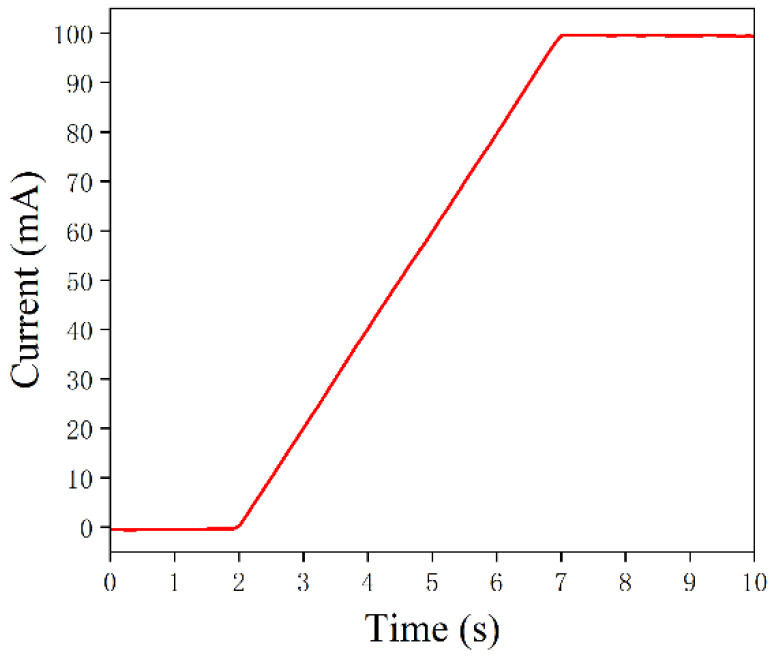
Test results of the slow-start circuit.

In addition to the current limiting and electrostatic protection circuits described in Section 3.3, the LD drive circuit requires a slow-start design so that the drive current increases gradually from zero to a set value, in order to avoid any inrush current damages when the LD is switched on and off [29]. In previous studies, resistor-capacitor (RC) slow-start circuits were usually used to suppress surge currents in LD driver circuits. The traditional RC slow-start circuit, which uses the capacitor charging and discharging principle, can eliminate the impacts of inrush currents. However, in the slow-start process of the RC slow-start circuit, the rate of increase in the voltage across the capacitor is not linear, but similar to a logarithmic function, which makes it difficult to accurately control the timing of the slow-start process [30]. This study proposed a DAC-based software-controlled linear slow-start strategy, which enables not only the precise control of the slow-start time, but also enables linear variation in the drive current. The operation principle is that when a semiconductor laser drive current is set, the microcontroller automatically calculates the corresponding set voltage $V_{i}$, and divides the set voltage $V_{i}$ equally to calculate the voltage increment $V_{inc}$. When the drive circuit is activated, the microcontroller controls the output voltage of the DAC for it to increase linearly from zero, each time by the value of $V_{inc}$, until the output voltage reaches the set voltage $V_{i}$. The slow-start circuit test results are shown in Figure 6, and it can be seen that the LD drive current linearly increased from 0 mA to 100 mA, with a slow-start time of 5 s. This strategy solves the problem of precisely controlling the slow-start time in traditional RC slow-start circuits, and achieves linear variation in the drive current. The slow-start time and the slope of the current change can also be flexibly adjusted by modifying the control parameters of the DAC software.

### 3.5. Temperature Control Circuit Design

A temperature measurement circuit consisting of an NTC thermistor can sense the temperature changes in the LD, and the temperature control of the LD can be realized by adjusting the magnitude and direction of the TEC current. The NTC thermistor $R_{TH}$ was connected in series with a detection resistor with an accuracy of 0.01% as part of a voltage divider circuit for proportional measurements. An NTC thermistor is a sensor resistance whose resistance value decreases with increasing temperature. When the temperature of the LD changes, the resistance value of the NTC thermistor will change accordingly, causing the voltage at both ends of the NTC thermistor in the series voltage divider circuit to change. Moreover, a constant excitation voltage is used to supply the voltage divider circuit, and ADC is used to collect the voltage across $R_{TH}$ in order to determine the resistance of $R_{TH}$, and thus the temperature of the LD. ADI’s AD7124 was chosen for the ADC2 in Figure 3. Using the AD7124’s internal reference voltage as the excitation voltage for the NTC thermistor and detection resistor, and using this voltage as a reference voltage for the AD7124 to perform measurements, the errors in the excitation voltage source could be eliminated, resulting in proportional measurement results.

**Figure 7:**
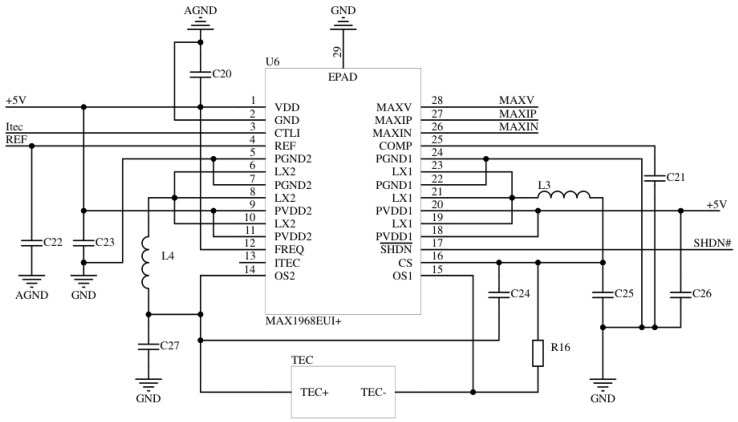
The TEC drive circuit.

The TEC operating state was controlled by MAX1968, a dedicated TEC driver chip. The direction and magnitude of the TEC drive current are determined by the control voltage magnitude of the MAX1968, and the relationship between the TEC drive current and control voltage is as follows:

(6) $$I_{TEC}=\frac{V_{CTLI}-V_{REF}}{10\cdot R_{SENSE}},$$

where $I_{TEC}$ is the TEC drive current, $V_{CTLI}$ is the control voltage of the MAX1968, $V_{REF}$ is the reference voltage (1.5 V), and $R_{SENSE}$ is the sense resistance of the TEC drive current (50 mΩ). The DAC was selected to replace the resistive voltage divider circuit, in order to control the voltage magnitudes of the MAXV, MAXIP, and MAXIN pins, allowing flexible settings for the maximum voltage, maximum forward current, and maximum reverse current across the TEC for its protection. Figure 7 shows the TEC drive circuit.

## 4. Implementation of the Temperature Control Algorithm

### 4.1. Model Construction of the Temperature Control System

Building reasonable and accurate system mathematical models helps to better understand control systems, and to enhance their performance. The NTC thermistor and TEC in the temperature control system both have thermal inertia, and the idealized mathematical model of the TEC can be regarded as a first-order inertia link [31]. It takes some time for the thermistor to sense temperature change in the LD, and some time for the feedback control of the TEC to manifest itself. Therefore, the semiconductor laser temperature control system can be regarded as a first-order inertia link object with an additional hysteresis link. Thus, the first-order inertia link to introduce a delay link is based on the transfer function, expressed as follows:

(7) $$G\left(s\right)=\frac{K}{Ts+1}e^{-\tau s},$$

where T is the time constant of TEC, K is the scaling factor, and τ is the pure lag time constant.

**Figure 8:**
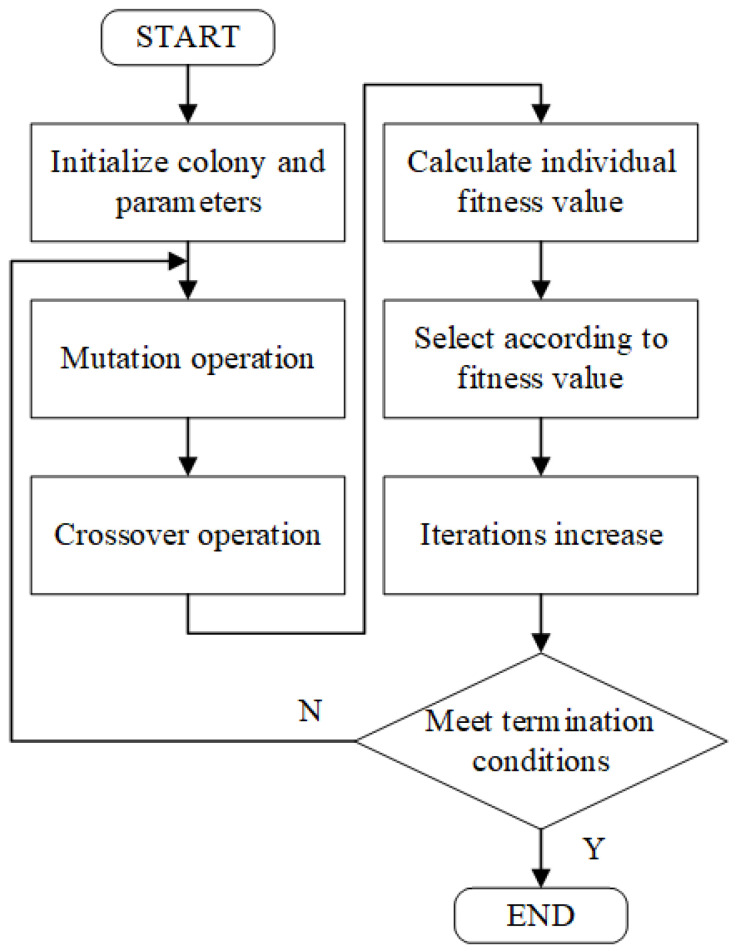
Flow chart of the differential evolution algorithm.

For the constructed mathematical model of the temperature control system, a model identification method based on the pseudo-random binary sequence and a DE algorithm was adopted to find the unknown quantities of T, K, and τ. The pseudo-random binary sequence, also known as the M sequence, is a sequence of random variables generated by a linear shift register that contains both “0” and “1” logic. The nature of the M sequence is close to that of white noise signals, and the principle is simple and easy to implement in engineering, as it can ensure that the system has good recognition accuracy [32]. The DE algorithm, as an emerging and evolutionary computational technique, has been used with success in a number of fields due to its simplicity, the low number of parameters to be determined, its fast convergence, and strong global search capabilities [33]. The generated M sequence was fed into the temperature control system as an excitation signal, and the logic “0” and “1” in the M sequence were converted into the corresponding TEC operating current. After the previous experiments and analysis, for the control system in this study, the order of the M sequence was set to 4 (i.e., the M sequence cycle period was 15), and the pulse width was set to 5 s, which was more reasonable. Meanwhile, the temperature change information of the LD was collected. When acquiring data, it should be noted that when the M sequence was first applied to the system, the output of the control system was non-stationary for a period of time, due to the non-zero initial conditions. To ensure the accuracy of identification, this non-stationary process should be avoided, and data can normally be obtained beginning from the second cycle of the M sequence. The unknown quantities of T, K, and τ were used as population individuals of the DE algorithm, setting the population size to 50 and the number of iterations G to 100. Figure 8 shows the solving process of the DE algorithm. The basic DE algorithm made the population diversity smaller as the number of evolutionary generations increased during the solution process, converged to local extremes prematurely, or caused the algorithm to stagnate. This was undoubtedly fatal to the DE algorithm that relied on population differences for evolution, and deteriorated the performance of the algorithm during the evolutionary process. In order to solve the mentioned drawbacks of the basic DE algorithm, Equations (8) and (9) were used to complete the mutation operation as well as the crossover operation for the characteristics of the DE algorithm, respectively, in order to avoid premature convergence of the DE algorithm to local extremes.

(8) $$h_{i}=X_{r1}+(F_{min}+0.5\cdot \left(1-\cos\left(i\cdot \pi /G\right)\right)\left(F_{max}-F_{min}\right)\left(X_{r2}-X_{r3}\right)$$

(9) $$\{\begin{array}{l} v_{i,j}=X_{i,j},rand\left(0,1\right)>\left(CR_{min}+0.5\cdot \left(1-\cos\left(i\cdot \pi /G\right)\right)\left(CR_{max}-CR_{min}\right)\right)\\ v_{i,j}=h_{i,j},else\;\;\;\;\;\;\;\;\;\;\;\;\;\;\;\;\;\;\;\;\;\;\;\;\;\;\;\;\;\;\;\;\;\;\;\;\;\;\;\;\;\;\;\;\;\;\;\;\;\;\;\;\;\;\;\;\;\;\;\;\;\;\;\;\;\;\;\;\;\;\;\;\;\;\;\;\;\;\;\;\;\;\;\;\;\;\;\;\;\;\;\;\;\;\;\;\;\;\;\;\;\;\;\;\;\;\;\;\;\;\;\;\;\;\;\;\;\;\; \end{array}$$

Equation (8) indicates that in the jth iteration, three individuals, $X_{r1}$, $X_{r2}$, and $X_{r3}$ (r1≠r2≠r3≠i), are randomly selected from the population, and the generated variance vector is $h_{i}$. The terms $F_{min}$ and $F_{max}$ stand for the minimum and maximum values of the scaling factor, which are generally chosen from [0,2]. The condition for the crossover operation on the population is shown in Equation (9). When the random number between 0 and 1 is greater than the set crossover condition, the crossover vector $v_{i,j}$ keeps the original vector value $X_{i,j}$ the same; otherwise, it is replaced with the generated variance vector $h_{i,j}$. In Equation (9), $CR_{min}$ and $CR_{max}$ denote the minimum and maximum values of the crossover operator, respectively, and the range of values is in [0,1].

**Figure 9:**
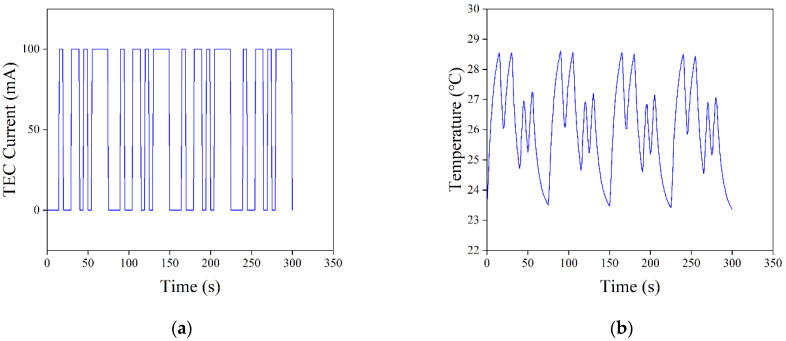
Excitation signal and output response of system identification: (**a**) excitation signal and (**b**) output response.

**Figure 10:**
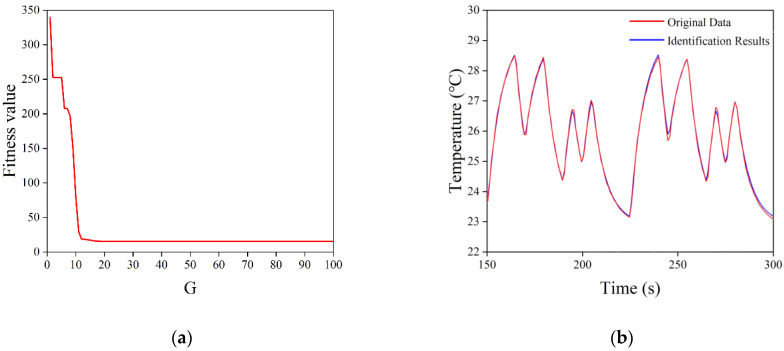
Identification results of the DE algorithm. (**a**) Change curve in the value of the fitness function. (**b**) Comparison curves of the identification results with the original data.

Based on the excitation signal and output response of the temperature control system, the model was identified using the DE algorithm to solve unknown parameters. The excitation signal and output response of the system identification are shown in Figure 9. The LD temperature varied with the TEC current, showing a steady periodic variation. As the number of iterations (G) increased, the fitness function converged rapidly to reach a minimum value of 15.41149 after 38 iterations, as shown in Figure 10a. The final transfer function of the temperature control system can be obtained as follows:

(10) $$G\left(s\right)=\frac{-0.061575}{7.1748s+1}e^{-0.412s}.$$

Based on the excitation signal, the corresponding system response was calculated using the obtained transfer function, and compared with the original data collected from the temperature control system. As shown in Figure 10b, the calculated values of the system response fit well with the collected raw data, indicating that the system functions obtained from the model identification scheme based on the M sequence and DE algorithms had a high degree of accuracy, and that it could be used for subsequent optimization of the control algorithm.

The system model constructed in this study demonstrated both applicability and high accuracy. For the laser temperature control system with a similar design structure as that in the study, the mathematical model given in Equation (7) could be directly applied. Only some parameters of the M sequence and DE algorithms needed to be modified according to the actual situation during the system identification process, in order to ensure a final system model with high accuracy. To ensure the accuracy of system identification, it is necessary to fully collect the input and output data of the system; however, a large amount of data leads to long computation times for the DE algorithm. It will be a future research direction for this method to reduce the computation time by optimizing the DE algorithm under the requirement that the accuracy of system identification will not be reduced.

### 4.2. Fuzzy PID Controller

**Figure 11:**
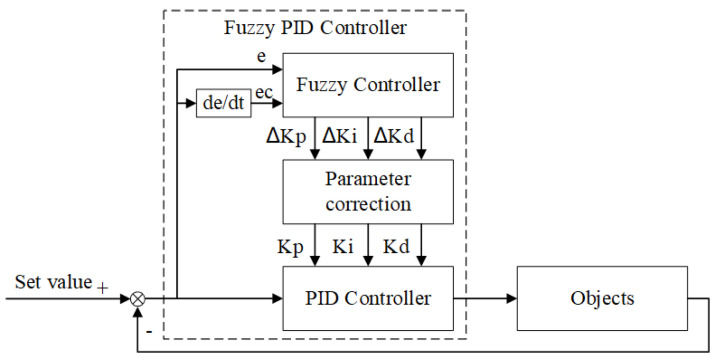
Schematic of the fuzzy PID controller.

Temperature control systems mostly use PID control algorithms. However, to improve the accuracy of temperature control due to complex interference factors, the PID parameters must be continuously adjusted during the control process [34]. Therefore, in this research, a fuzzy PID controller was chosen as a temperature control algorithm; it could compensate for accuracy shortcomings of fuzzy control and realize the real-time adjustment of the control parameters of the PID algorithm. The working principle of the fuzzy PID controller is shown in Figure 11. The fuzzy PID controller takes the error e and the error rate of change ec as inputs, and adjusts the proportional coefficient $K_{p}$, integral coefficient $K_{i}$, and differential coefficient $K_{d}$ of the PID algorithm according to the set fuzzy control rules in order to meet requirements of the control parameters at different e and ec. Thus, control systems could have good dynamic and static performance.

According to the actual condition of the semiconductor laser temperature control system, the intervals of e, ec, $K_{p}$, $K_{i}$, and $K_{d}$ were set as [−15,15], [−5,5], [−5,5], [−2,2], and [−2,2], respectively, and the fuzzy subsets of the input and output parameters were all set as follows: {negative big, negative medium, negative small, zero, positive small, positive medium, positive big} = {NB, NM, NS, ZO, PS, PM, PB}. Moreover, the affiliation function was triangular, and the centroid method was chosen for clarity. The centroid method takes the center of gravity of the area enclosed by the affiliation function curve and the horizontal coordinate as the final output value of the fuzzy controller. For the case of a discrete domain with m output quantization levels, we use the following:

(11) $$u=\frac{\sum_{k=1}^{m}v_{k}\mu_{v}\left(v_{k}\right)}{\sum_{k=1}^{m}\mu_{v}\left(v_{k}\right)},\;$$

where u is the exact value of the fuzzy controller output after defuzzification, $v_{k}$ is the value in the domain of the fuzzy control quantity, and $\mu_{v}\left(v_{k}\right)$ is the affiliation value of $v_{k}$. The centroid method has smoother output control, and the output will change even if it corresponds to a small change in the input signal. The $\Delta K_{p}$, $\Delta K_{i}$, and $\Delta K_{d}$ in Figure 11 are the exact output values of the fuzzy controller after defuzzification by the centroid method. The new control parameters were obtained by adding the corresponding corrections $\Delta K_{p}$, $\Delta K_{i}$, and $\Delta K_{d}$ to the original control parameters $K_{p}$, $K_{i}$, and $K_{d}$, in order to achieve real-time modification of the control parameters of the PID controller. The final control signal was obtained by the incremental PID calculation shown in Equation (12):

(12) $$\Delta u\left(k\right)=K_{p}\left[e\left(k\right)-e\left(k-1\right)\right]+K_{i}e\left(k\right)+K_{d}\left[e\left(k\right)-2e\left(k-1\right)+e\left(k-2\right)\right],\;$$

where Δu(k) denotes the kth control signal increment, and e(k), e(k−1), and e(k−2) represent the error values of the kth, k−1, and k−2 times, respectively.

**Figure 12:**
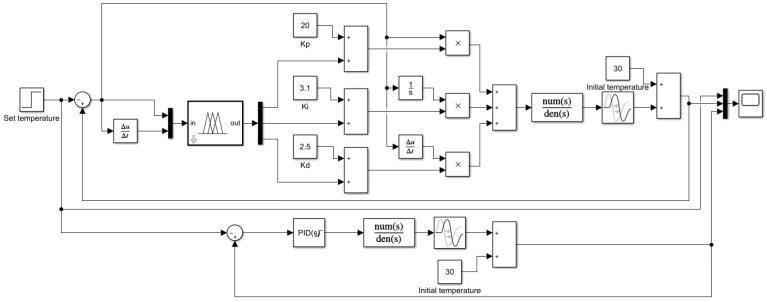
MATLAB/Simulink system simulation.

**Figure 13:**
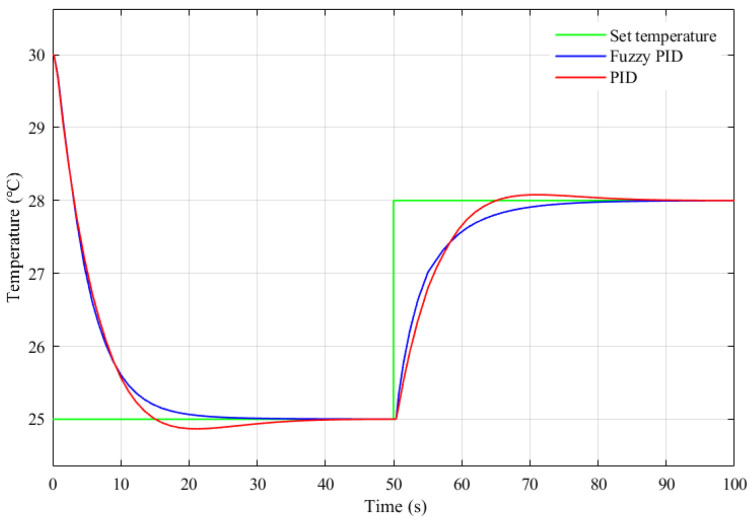
Simulation comparison of control effects.

The fuzzy control rules at the core of a fuzzy controller are not specific to a particular mathematical model, and can be applied to a wide range of control systems. To improve the performance of the temperature control system, the fuzzy control rules were simulated and optimized for the temperature control system designed in this study. The fuzzy control rules that were formed from previous experiences were used as the initial fuzzy control rules. Simulink is a software package for modeling, simulation, and analysis of dynamic systems integrated in MATLAB, which has powerful modeling capabilities and visualization functions and has been widely used in the simulation and design of control systems [35]. According to the mathematical model constructed in Section 4.1, Simulink was used to build the simulation system shown in Figure 12, and also to obtain its input and output data. According to the response of the system, the initial fuzzy control rules were iteratively adjusted using the MATLAB fuzzy toolbox, and the final fuzzy control rules were obtained, as shown in Table 1. The optimized fuzzy PID controller was simulated using Simulink to compare with the regular PID controller, and the simulation results as shown in Figure 13. As can be seen from Figure 13, compared with the regular PID controller, the optimized fuzzy PID controller had two advantages: the response was more rapid when the set value was changed, and the overshoot was reduced; moreover, the change was smoother near the set value, avoiding frequent oscillations.

## 5. Experimental Results and Analysis

**Figure 14:**
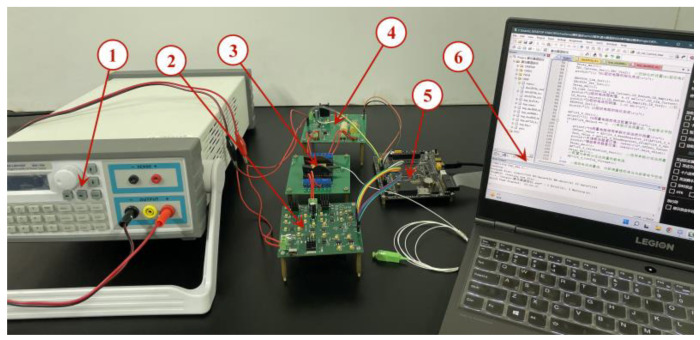
Semiconductor laser current drive and temperature control experimental system: (1) DC regulated power supply, (2) constant-current driver circuit, (3) butterfly semiconductor laser, (4) temperature control circuit, (5) microcontroller, and (6) PC.

The semiconductor laser current drive and temperature control system designed in this study is shown in Figure 14. The system is powered by a direct current (DC) regulated power supply, and the personal computer (PC) collects and displays the system operation information in real time. The experiments were conducted at 27 °C in an indoor environment without significant ventilation.

### 5.1. Experiments with Constant-Current Output Characteristics

**Figure 15:**
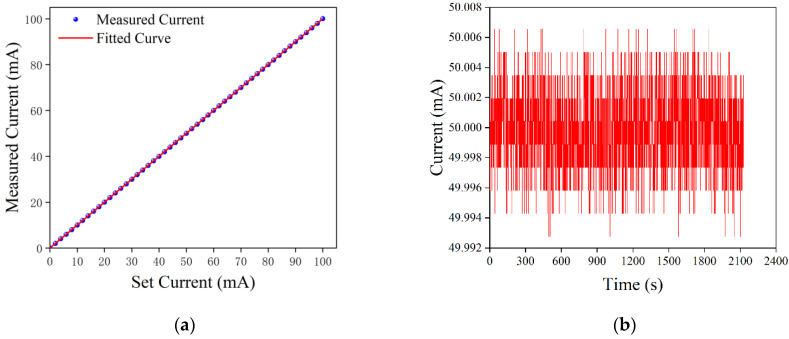
Test results of the drive current: (**a**) linearity of the drive current and (**b**) stability of the drive current.

The driving current test range was set from 0 to 100 mA, and the driving current was set at 2 mA intervals to obtain the relationship between the actual and set values of the driving current. Based on the experimental data, the actual value of the drive current, $I_{LD}$, was fitted to the set value, $I_{SET}$, using the least squares method, and the fitted curve was demonstrated, as shown in Figure 15a. Its first-order fitting equation is as follows:

(13) $$I_{LD}=0.99981\times I_{SET}-0.15211.$$

Using the linearity formula, as follows:

(14) $$\gamma_{1}=\left(\left|\Delta I_{max}\right|/I_{max}\right)\times 100\%,$$

the linearity between the set and actual values of the drive current was found to be 0.0129%. Moreover, the maximum deviation $\Delta I_{max}$ between the actual and set values of the drive current was 0.0129 mA, which occurred at 14 mA, and the maximum current setting value $I_{max}$ was 100 mA.

The drive current stability was tested by setting the drive current to 50 mA, and the test results were demonstrated, as shown in Figure 15b. During the test time period, the maximum value of the drive current was 50.0065 mA, the minimum value was 49.9928 mA, and the average value was 49.9998 mA. Using the following stability equation, the drive current stability was found to be ~0.0274%, where $I_{0}$ is the drive current setting value:

(15) $$\gamma_{2}=\left[\left(I_{max}-I_{min}\right)/I_{0}\right]\times 100\%.$$

The laser operating current could be kept stable for a long time, which helped to improve the stability of the output wavelength and optical power of the semiconductor laser. In practical applications, the linearity between the input and output of the drive circuit will become worse due to the influence of instability factors, such as the deviation of the components themselves and the ambient temperature. By fully differentiating Equation 5 and substituting specific values, the degree of influence of each part of the drive current control loop on the current stability can be calculated. The stability of the drive current is mainly affected by the fluctuation of the reference voltage and the temperature drift of the sampling resistor. In order to obtain a more stable drive current, it is necessary to continue improving the stability of the reference voltage, and to use a sampling resistor with a smaller temperature drift.

### 5.2. Temperature Control Experiments with Semiconductor Lasers

**Figure 16:**
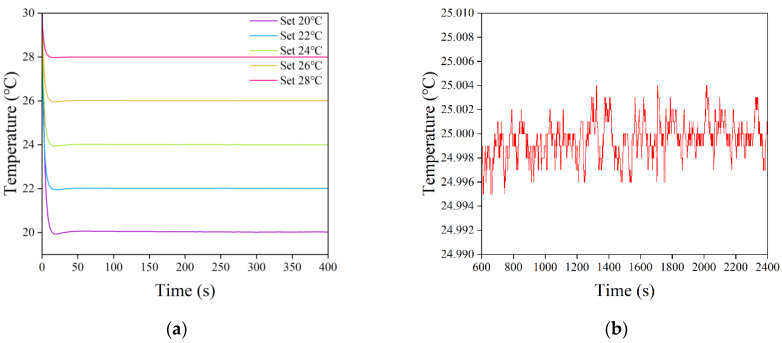
Temperature variation curve of a semiconductor laser: (**a**) temperature control stability experiment and (**b**) temperature control accuracy experiment.

The semiconductor laser drive current was set to 50 mA, and the initial operating temperature was set to 30 °C. After the laser operating temperature was stabilized, the set temperature was changed to 20 °C, 22 °C, 24 °C, 26 °C, and 28 °C, in turn, to test the temperature control stability. As shown in Figure 16a, after the set temperature was changed, the LD temperature rapidly approached the target value, reached the temperature minimum in ~20 s, and gradually stabilized around the target value after a small overshoot, where the maximum overshoot was ~0.07 °C. As the difference between the set temperature value and initial operating temperature increased, the overshoot of the temperature profile slowly increased, and the time taken for the temperature profile to stabilize around the set value also accordingly increased. The drive current setting was kept constant, and the laser operating temperature was set to 25 °C. After the LD temperature was stabilized, the continuous change data were intercepted for half an hour to observe the temperature control accuracy. As shown in Figure 16b, the actual operating temperature of the LD fluctuated up and down around the set temperature, and the temperature fluctuation was small, with a minimum temperature of 24.995 °C, a maximum temperature of 25.004 °C, and a maximum temperature error of 0.005 °C, thus exhibiting high temperature control accuracy.

## 6. Conclusions

In this study, a high-precision semiconductor laser current drive and temperature control system was proposed on the basis of analyses performed on semiconductor laser characteristics, hardware circuit design, control system mathematical model construction, and control algorithm simulation and optimization. Closed-loop negative feedback control was used, and it reduced current fluctuations and improved the linearity and stability of the drive current. The DAC-based software-controlled slow-start strategy overcame the disadvantages of conventional RC slow-start circuits, such as imprecise delay times, while realizing a linear variation in the drive current. Moreover, the M sequence and DE algorithm combined with the system identification scheme was able to accurately model the temperature control system and provide an important basis for the optimization of control algorithms. A fuzzy PID controller was used to adjust the PID control parameters online, and the fuzzy control rules were optimized through system simulation to make the fuzzy controller more suitable for the temperature control system of the semiconductor laser designed in this research. The experimental results showed that the system achieved a linear adjustable drive current within 0–100 mA, with a linearity of 0.0129% and a stability of 0.0274%, and that it showed good temperature control stability with a temperature control accuracy of 0.005 °C.

Overall, the current drive and temperature control system designed in this study can be used in applications that require high laser output stability, and it can also be used as a reference for the design of current drive and temperature control systems for semiconductor lasers.

## Figures and Tables

**Table 1:** Table of the fuzzy control rules ($\Delta K_{p}$/$\Delta K_{i}$/$\Delta K_{d}$).

E	EC						
	NB	NM	NS	ZO	PS	PM	PB
NB	PB/NB/PS	PB/NB/NS	PM/NM/NB	PM/NM/NB	PS/NS/NB	ZO/ZO/NM	ZO/ZO/PS
NM	PB/NB/PS	PB/NB/NS	PM/NM/NB	PS/NS/NM	PS/NS/NM	ZO/ZO/NS	NS/ZO/ZO
NS	PM/NB/ZO	PM/NM/NS	PM/NS/NM	PS/NS/NM	ZO/ZO/NS	NS/PS/NS	NS/PS/ZO
ZO	PM/NM/ZO	PM/NM/NS	PS/NS/NS	ZO/ZO/NS	NS/PS/NS	NM/PM/NS	NM/PM/ZO
PS	PS/NM/ZO	PS/NS/ZO	ZO/ZO/ZO	NS/PS/ZO	NS/PS/ZO	NM/PM/ZO	NM/PB/ZO
PM	PS/ZO/PB	ZO/ZO/PS	NS/PS/PS	NM/PS/PS	NM/PM/PS	NM/PB/PS	NB/PB/PB
PB	ZO/ZO/PB	ZO/ZO/PM	NM/PS/PM	NM/PM/PM	NM/PM/PS	NB/PB/PS	NB/PB/PB

## Data Availability

Not applicable.
